# In-Orbit Performance Evaluation of a Spaceborne High Precision Fiber Optic Gyroscope

**DOI:** 10.3390/s18010106

**Published:** 2018-01-01

**Authors:** Jing Jin, Ting Zhang, Linghai Kong, Kun Ma

**Affiliations:** Department of Opto-Electronics Engineering, Beihang University, Beijing 100191, China; jinjing@buaa.edu.cn (J.J.); konglinghai1990@163.com (L.K.); kunma@buaa.edu.cn (K.M.)

**Keywords:** fiber optic gyroscope, in-orbit performance evaluation, random walk coefficient

## Abstract

An in-orbit experiment was launched to evaluate the performance of the spaceborne high precision fiber optic gyroscopes (FOG). The three-axis in-orbit data of the FOG were analyzed using wavelet analysis method. Features of low frequency period terms and glitch noise were demonstrated. In addition, a method to extract the random noise from the in-orbit data is proposed based on the first-order difference method and the Pauta criterion. In addition, the random walk coefficient (RWC) of the FOG was calculated with the Allan variance method. Compared the ground test results, the in-orbit performance evaluation of Spaceborne High Precision Fiber Optic Gyroscope was verified.

## 1. Introduction

With the increase of gyroscope space applications, people put forward higher requirements on the performance of the gyroscope. The Gyroscope is moving toward to the directions of high precision, low cost and miniaturization. Because of its cost performance and lifetime, the electromechanical gyroscope is gradually replaced by the optical gyroscope. The ring laser gyroscope (RLG) has made great progress in the accuracy and reliability, but its mechanical dither device to solve the lock-in effect makes its system complex and cannot be applied in space [1]. Moreover, the resonant micro optical gyroscope (RMOG), which is considered to be the ideal candidate for optoelectronic gyroscopes miniaturization, is still in the laboratory research stage [2,3]. In addition, the fiber optic gyroscope (FOG) began to be used in space due to its high reliability, long life, small volume, high precision and no moving parts [4,5,6,7,8,9,10]. The FOG is the best choice for space applications at this stage. This makes it even more important to evaluate the performance of the FOG served in space mission.

There are the intrinsic random noise and bias drift involved in FOG output data [11,12,13,14,15]. When the FOG fails or the performance degrades, the random noise will change significantly. In addition, the random walk coefficient (RWC) is an important technical indicator to assess random noise [16]. In this paper, we designed an in-orbit experiment evaluating the performance of the FOG. Features of acquired data were found by wavelet analysis. An extraction method developed to get the random noise, and the estimated RWC was calculated using the in-orbit data.

## 2. In-Orbit Performance Evaluation Experiment Setup

**Figure 1:**
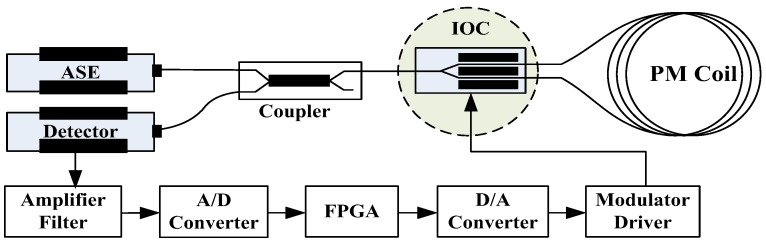
The structure diagram of the experimental FOG.

An in-orbit experiment was conducted to evaluate the performance of the spaceborne high-precision fiber optic gyroscopes. The experimental FOG is a three-axis structure which can provide three-dimensional angular rate information. One axis structure diagram is shown in Figure 1. This picture shows the typical configuration of the high precision FOG. The operating wavelength of the amplified spontaneous emission (ASE) source is 1550 nm, and the length of the polarization maintaining (PM) coil is 1000 m. The results of ground test of the FOG are shown in Table 1.

**Figure 2:**
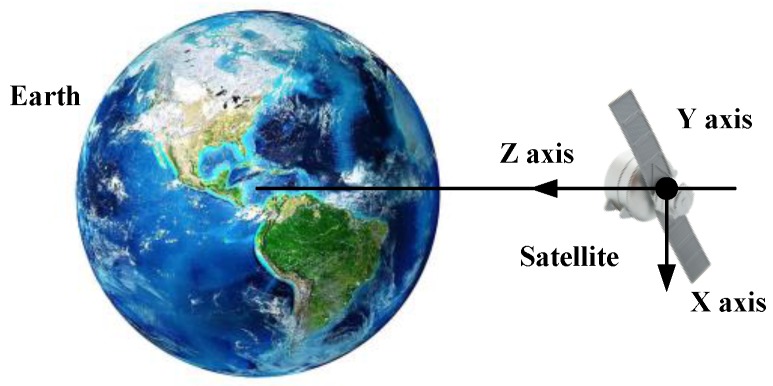
The experimental FOG attitude related to earth.

**Figure 3:**
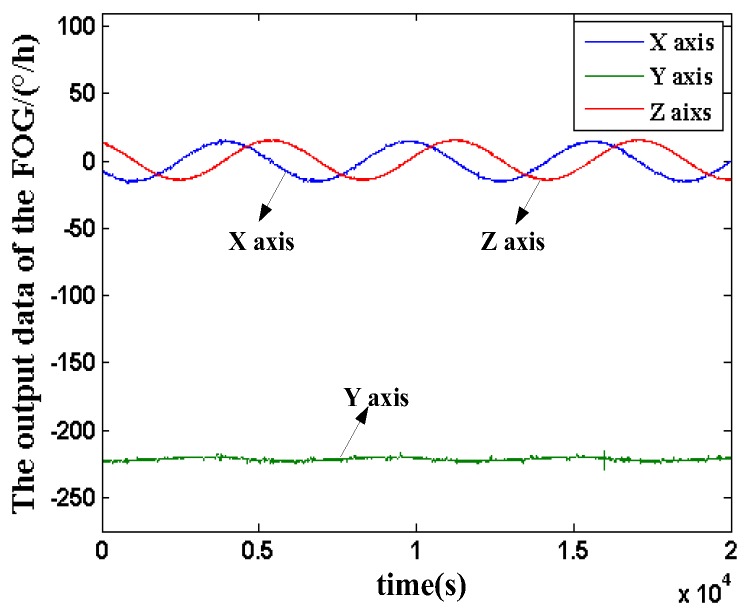
The output data of the three-axis FOG.

The FOG was mounted on the new science exploration and technology demonstration satellite. The satellite is mainly used for the tests of the satellite high-precision, high performance, components, satellite formation and inter-satellite measurement and link and in-orbit verification of the new principles, new technologies, new equipment and materials required for satellite development. The attitude of the experimental three-axis FOG mounted on the satellite is shown in Figure 2. *X*, *Y* and *Z* indicate the axial direction. The *Z* axis points to the earth. The *X* axis is in the moving direction, which is parallel to the orbital plane. In addition, the *Y* axis is perpendicular to the orbital plane. The three axes in the FOG are orthogonal to each other and axially aligned with the satellite. The Satellite is in the sun-synchronous orbit with the height of 645 km and the orbit inclination of 98°. The time of the satellite orbiting the Earth a week is about 98 min and the average angular rate is −219.6°/h. During the evaluation experiments in one year, there is no maneuvers were commanded Through the monitoring of the telemetry data, the 20 sets three-axis in-orbit output data of the FOG were acquired in one year and the typical nineteenth group are taken as an example shown in Figure 3.

## 3. Analysis of In-Orbit FOG Data

The average angular velocity of the *Y* axis is −221.5°/h and the output data of *X*axis and *Z* axis are very small. It can be seen that the three-axis output data of the experimental FOG is consistent with the estimation on ground. In addition, the three-axis in-orbit data have the similar characteristics and the data of *Y* axis is typical. Thus, the *Y* axis data was taken as a representative for data analysis and processing.

**Figure 4:**
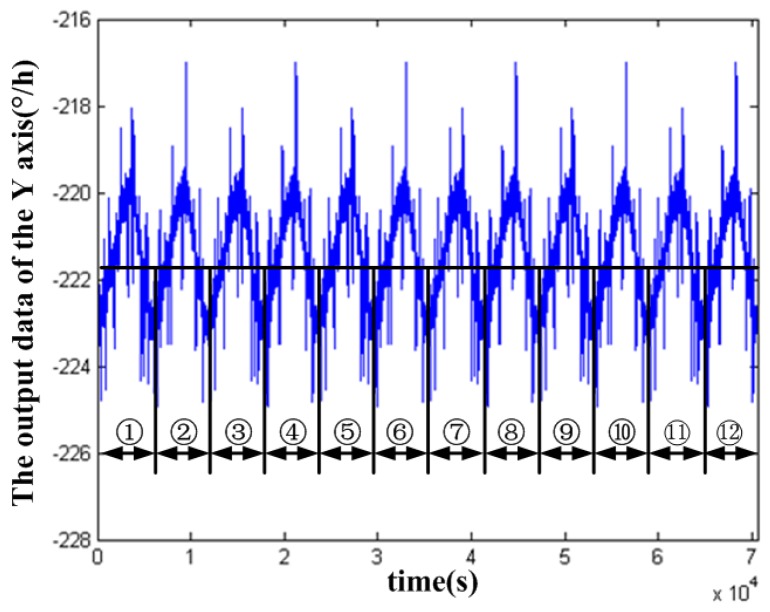
Division of data for calculating the bias instability.

Twelve cycles *Y* axis acquired in-orbit data in the nineteenth group was drawn in Figure 4 and each cycle lasts for 5580 s. First calculate the mean value of each cycle output data, then the bias instability of the in-orbit data was obtained by calculating the standard deviation of the mean value. It was calculated that the bias instability of the *Y* axis data was 0.0029°/h. Then, the same method was used to calculate the bias instability of the 20 sets three-axis data of the FOG as shown in Table 2, which are all smaller than the application requirement of 0.01°/h, and the mean value of the three-axis output data of the FOG is stable and the fluctuation is small. So it can be determined that the satellite attitude is stable.

**Figure 5:**
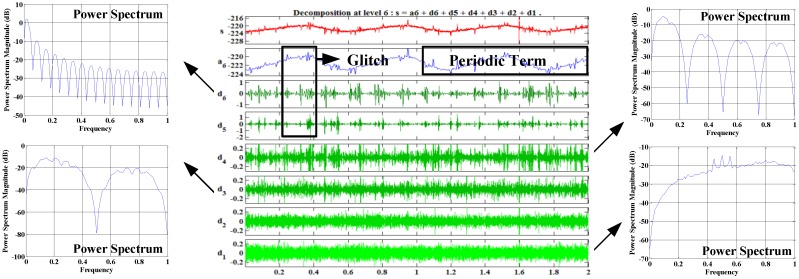
*Y* axis data characteristics demonstrated by wavelet analysis.

The *Y* axis data is decomposed by Daubechies 4 orders wavelet transform [17], which is shown in Figure 5. Through the wavelet analysis, it can be seen that there are obvious low-frequency period items in the in-orbit data, which is due to the FOG perceiving the rotation of the satellite around the earth. Besides, the glitches in the in-orbit data are mainly due to the adjustment of the satellite attitude. Other noise is caused by the internal and external environment of the FOG.

Analyzing the characteristics of each band of the data and drawing the power spectrum of each detail of the wavelet decomposition as shown in Figure 5, it can be seen that the data has a considerable smoothness in high frequency band and obvious fluctuation in low frequency band.

**Figure 6:**
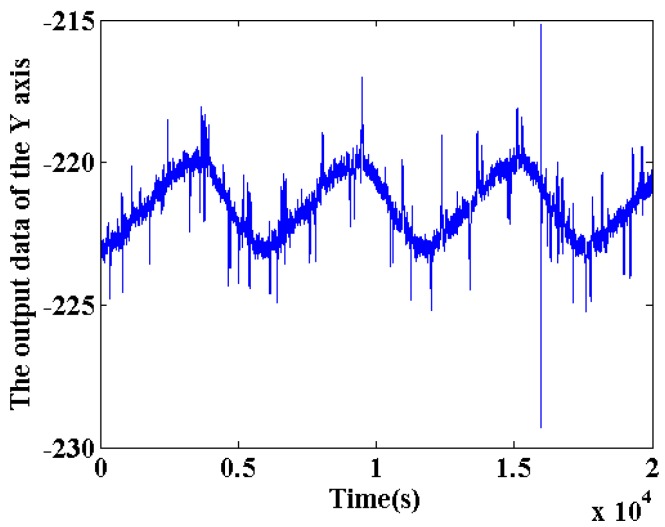
The output data of the *Y* axis in 19th set.

**Figure 7:**
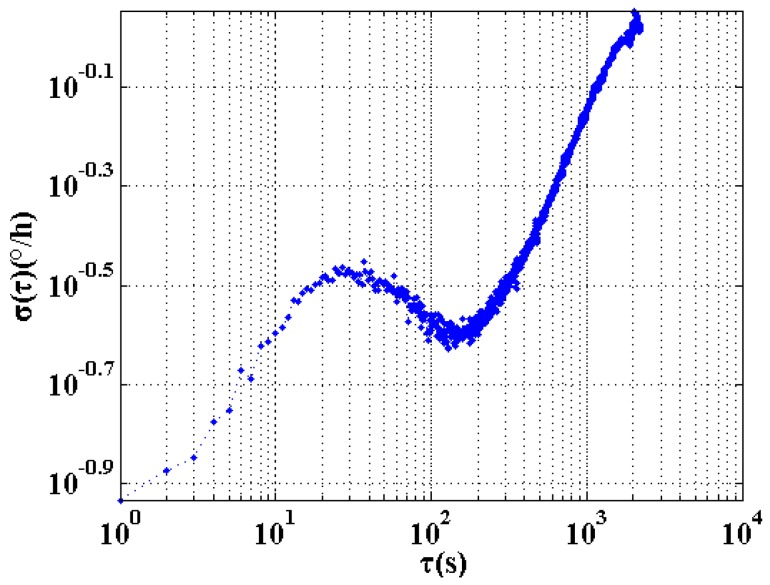
Allan variance curve of the *Y* axis output data.

The Allan variance method is used to process the data collected in orbit, and the values of the RWCs are obtained by fitting. Therefore, the selected *Y* axis output data as shown in Figure 6 from 19th set with strong data characteristics was calculated by Allan variance method directly, the results shown in Figure 7.

The Allan Variance method is used to analyze the characteristics of noise in fiber optic gyros. When the significant trend of low-frequency period exists in the raw in-orbit data, the Allan variance curve fluctuates greatly and therefore RWC cannot be correctly calculated. Therefore, it is necessary to perform data processing to extract random noise in orbit data.

## 4. Extraction of Random Noise of the In-Orbit FOG

**Figure 8:**
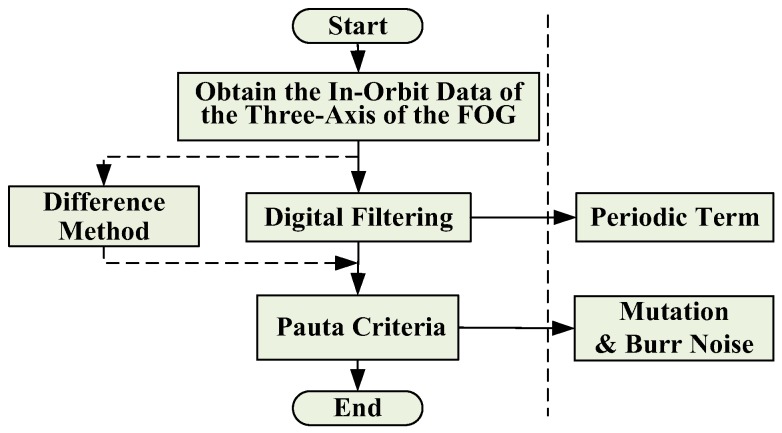
In-orbit data processing flow chart.

In order to obtain the random noise from the in-orbit data, the data processing will be carried out from the viewpoint of removing the disturbance margin, mainly through the digital filtering and difference method to remove the low frequency period of the data. In addition, the glitch noise in the data is removed according to the Pauta criterion. The flow chart of the process is shown in Figure 8.

**Figure 9:**
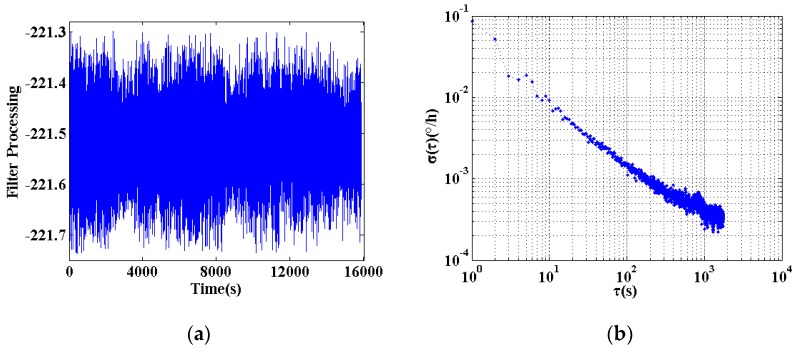
In-orbit data digital filtering processing. (**a**) Data after digital filtering; (**b**) Allan variance of the data after digital filter.

From the above data analysis shows that compared with the low frequency band, in-orbit data collected at high frequencies has a more stable characteristic. Therefore, this study chooses the cutoff frequency to be the half of the maximum frequency of the data, i.e., 0.25 Hz. The high-pass filter is used to remove the low-frequency period terms, and then the mutation and the glitch noise are removed according to the Pauta criterion [18]. The Pauta criterion first assumes that a set of test data contains only random errors, and calculates the standard deviation of the data. By a probability of 99.7% to determine a range of 3 times standard deviation, that any error exceeds this range is not a random error but a gross error, the data containing the error should be removed. The processing result is shown in Figure 9.

In addition, the study shows that using the first-order difference method instead of digital filtering can also achieve the same accuracy of the processing effect. The first-order difference is the difference between adjacent two terms in a discrete function. Define a sequence *X*(*i*), then the first-order difference function can be expressed as Equation (1).

(1) $$Y_{1}(i)=X(i+1)-X(i),$$

**Figure 10:**
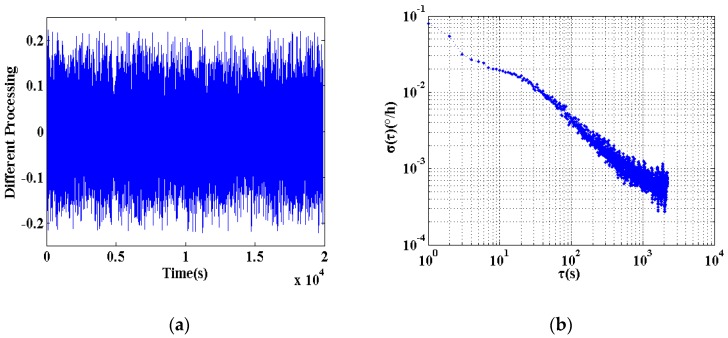
In-orbit data difference method processing. (**a**) Data after processing with difference method; (**b**) Allan variance of the data after processed with difference method.

The result of the data processing was shown in Figure 10.

The RWCs were calculated by Allan variance method for the three-axis data of the group, which were processed by the above two methods. The calculation results are shown in Table 3. It can be seen that the first order difference method can replace the digital filter method, and the calculation speed is faster.

The data processed by this study have good smoothness in the high frequency range, so the data can be processed by the method of data filtering or first order difference method combined with the Pauta criterion. However, when there is still a significant fluctuation in the high frequency of the data, the digital filtering method cannot meet the processing requirements, as the high frequency data fluctuation and the glitch noise cannot be removed. Therefore, only the difference method can be used to complete the data processing. Therefore, the follow-up data processing in this study mainly adopts the data processing method combined with first-order difference and Pauta criterion.

## 5. FOG Performance Evaluation and Verification

The Allan variance method is an important means of measuring and evaluating various types of error and noise characteristics of FOGs. The Allan variance method can be used to obtain the characteristics of various noise items in gyro data. It is an important value for the characterization of optical gyro noise. According to IEEE Std. 952™-1997 (R2008), FOG noise mainly includes quantization noise, angular random walk, zero partial instability, rate random walk and rate slope. In addition, the respective noise coefficients Q, N, B, K, and R are used to represent their sizes, respectively.

Allan variance reflects the fluctuation of the average frequency difference in the adjacent two samples, which is related to the bilateral power spectral density (PSD) $S_{\omega }(f)$ of the noise term in the raw measurement data:

(2) $$\sigma_{A}^{2}(\tau )=4\int_{0}^{\infty }S_{\omega }(f)\frac{\sin^{4}(\pi f\hat{\tau })}{{\left(\pi f\hat{\tau }\right)}^{2}}df,$$

On the formula: When passing through a filter with a transfer function $\sin^{4}(\pi f\hat{\tau })/{\left(\pi f\hat{\tau }\right)}^{2}$, Allan variance is proportional to the total energy of the noise output from the gyro. Different types of stochastic processes can be checked by adjusting the filter band-pass $\hat{\tau }$.

If the noise sources are statistically independent, the Allan variance can be expressed as follows:

(3) $$\sigma^{2}(\tau )=\sigma_{Q}^{2}(\tau )+\sigma_{N}^{2}(\tau )+\sigma_{B}^{2}(\tau )+\sigma_{K}^{2}(\tau )+\sigma_{R}^{2}(\tau ),$$

(4) $$\sigma^{2}(\tau )=\frac{3Q^{2}}{\tau^{2}}+\frac{N^{2}}{\tau }+{\left(0.6648B\right)}^{2}+\frac{K^{2}\tau }{3}+\frac{R^{2}\tau^{2}}{2}=\sum_{n=-2}^{2}A_{n}\tau^{n},$$

A least squares method is used to fit the curve of $\sigma^{2}(\tau )$, and the relationship between the coefficient An and the error coefficient is obtained:

Where N is the RWC.

**Figure 11:**
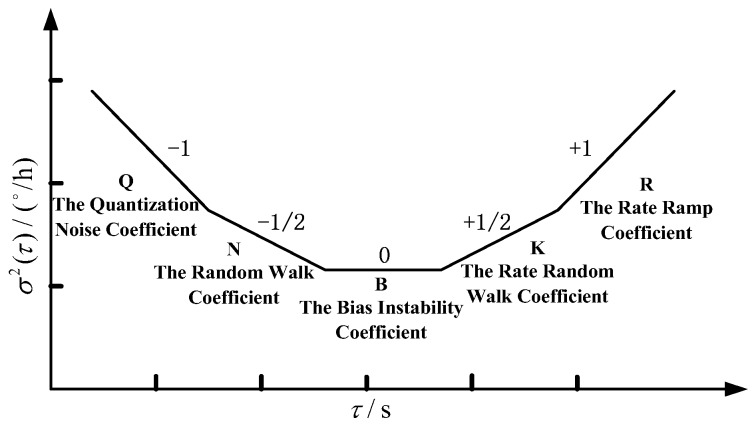
Typical FOG Allan variance graph.

According to the Allan variance σ(τ)−τ double logarithmic curve of the output FOG data shown in Figure 11, the coefficient is calculated to determine whether the performance of the gyroscope meets the requirements of its system.

**Figure 12:**
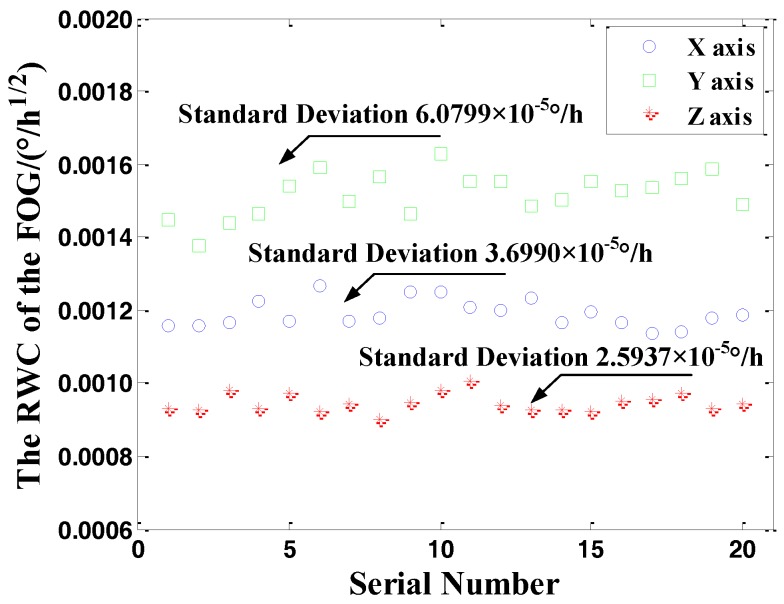
Three-axis RWC of the FOG in one year.

During the test period of the FOG, the three-axis output data of the FOG was collected 20 times in one year. The data processing was carried out by using the above method and the RWC was calculated by Allan variance method. The RWC curve is drawn in Figure 12.

The above data processing method is further verified by the processing of the remaining 19 sets of FOG data collected in orbit. The results show that this method. This method is effective under the condition of satellite attitude stabilization, and the random noise in the data can be extracted. In order to complete the performance evaluation of the experimental FOG, the RWC in-orbit calculations and ground test results are compared in Table 4.

In this study, the RWCs of the in-orbit FOG were calculated and the fluctuation of the RWC during the year was demonstrated by its variation curve. It can be seen that the standard deviation of RWC is small, so it can be confirmed that the in-orbit output data is stable. The RWC results of the *X* axis and *Z* axis are basically the same as those of the ground test. The RWC of the *Y* axis is slightly larger than that of the ground due to the sensitivity of the satellite attitude adjustment. However, the calculation results of three axes are all less than the performance index of the optical fiber gyroscope 0.002°/h. Therefore, it can be determined that the working state of the optical fiber gyroscope is stable and normal.

## 6. Conclusions

An in-orbit experiment was developed to evaluate the performance of the spaceborne high precision fiber optic gyroscopes. The analysis shows that the acquired in-orbit data contain the low-frequency period term due to the satellite cycle rotation and the mutation and glitch noise caused by the satellite attitude adjustment. The raw data cannot obtain the RWC due to the disturbance of the low-frequency trend. Then, a data processing method based on first order difference and Pauta criterion to extract noise is proposed. Based on the Allan variance method, the RWC of the FOG is calculated. The mean value of RWC of three axes is 1.417 × 10^−3^°/h^1/2^, which is match with the ground test result of 1.144 × 10^−3^°/h^1/2^. Thence, it can be determined that the performance of the in-orbit experimental FOG is stable and normal and the data analysis and extraction methods are feasible.

## Figures and Tables

**Table 1:** Performance of the experimental FOG.

Bias Instability	Scale Factor Error	Random Walk Coefficient	Output Range
≤0.01°/h	≤20 ppm	≤0.002°/h^1/2^	±20°/s

**Table 2:** The bias instability calculated with the 20 sets in-orbit data.

No.	*X* Axis (°/h)	*Y* Axis (°/h)	*Z* Axis (°/h)	No.	*X* Axis (°/h)	*Y* Axis(°/h)	*Z* Axis (°/h)
1	0.0042	0.0072	0.0025	11	0.0052	0.0091	0.0050
2	0.0045	0.0095	0.0018	12	0.0044	0.0062	0.0013
3	0.0068	0.0075	0.0049	13	0.0052	0.0079	0.0028
4	0.0070	0.0070	0.0099	14	0.0039	0.0066	0.0070
5	0.0041	0.0052	0.0015	15	0.0080	0.0060	0.0083
6	0.0095	0.0062	0.0016	16	0.0018	0.0050	0.0018
7	0.0057	0.0054	0.0096	17	0.0063	0.0070	0.0018
8	0.0057	0.0085	0.0028	18	0.0028	0.0084	0.0022
9	0.0049	0.0066	0.0053	19	0.0022	0.0029	0.0020
10	0.0058	0.0074	0.0025	20	0.0024	0.0063	0.0028

**Table 3:** The three-axis RWCs Calculated with Allan Variance Method.

	*X* Axis (°/h^1/2^)	*Y* Axis (°/h^1/2^)	*Z* Axis (°/h^1/2^)
Digital filtering	0.00134560	0.00182747	0.00107258
First order difference	0.00137787	0.00178724	0.00113209

**Table 4:** The RWC fluctuations of the three-axis FOG output data.

	*X* Axis (°/h^1/2^)	*Y* Axis (°/h^1/2^)	*Z* Axis (°/h^1/2^)	Mean Value (°/h^1/2^)
In-Orbit Test	1.389 × 10^−3^	1.718 × 10^−3^	1.145 × 10^−3^	1.417 × 10^−3^
Ground Test	1.133 × 10^−3^	1.183 × 10^−3^	1.117 × 10^−3^	1.144 × 10^−3^
